# Case Report: Composite Angioimmunoblastic T-Cell Lymphoma and Epstein-Barr Virus-Positive B-Cell Lymphoproliferative Disorder as Other Iatrogenic Immunodeficiency-Associated Lymphoproliferative Disorders

**DOI:** 10.3389/fmed.2020.625442

**Published:** 2020-12-23

**Authors:** Seiji Kakiuchi, Kimikazu Yakushijin, Ikumi Takagi, Junpei Rikitake, Hiroaki Akiyama, Hiroyuki Matsuba, Yoshitake Hayashi, Kazuyoshi Kajimoto, Nobuko Iwata

**Affiliations:** ^1^Department of Hematology, Yodogawa Christian Hospital, Osaka, Japan; ^2^Division of Medical Oncology and Hematology, Kobe University Graduate School of Medicine, Kobe, Japan; ^3^Division of Molecular Medicine & Medical Genetics, Department of Pathology, Kobe Graduate School of Medicine, Kobe, Japan; ^4^Department of Pathology, Hyogo Cancer Center, Akashi, Japan

**Keywords:** composite lymphoma, Epstein-Barr virus reactivation, methotrexate, angioimmunoblastic T-cell lymphoma (AITL), clonal expansion

## Abstract

Immunosuppressants are widely used to treat patients with rheumatoid arthritis (RA), and their adverse effects have been known to cause other iatrogenic immunodeficiency-associated lymphoproliferative disorders (OIIA-LPDs). We report a patient with RA who had been treated with methotrexate (MTX) and tacrolimus (TAC) and who developed whole body lymphadenopathy. We simultaneously confirmed angioimmunoblastic T-cell lymphoma (AITL) through a right cervical lymph node biopsy and Epstein-Barr virus-positive B-cell lymphoproliferative disorder (EBV-positive B-LPD) through a bone marrow examination. After cessation of immunosuppressant therapy, both LPDs completely disappeared. Patients with AITL are occasionally reported to develop B-cell lymphoma through reactivation of the EBV, which leads to clonal expansion in the microenvironment. Immunohistochemistry results revealed that both LPD components were positive for EBV-encoded RNA. Moreover, in this patient, the plasma EBV DNA level was found to be high; therefore, EBV infection was a probable etiology. Synchronous coexistence of AITL and B-LPD as an OIIA-LPD has rarely been reported. This case report is the first to discuss the disappearance of both LPDs on withdrawal of immunosuppressants only. AITL occasionally accompany B-LPD; however, this composite lymphoma comprised AITL and B-LPD, and OIIA-LPDs should not be overlooked.

## Introduction

According to the World Health Organization classification of tumors of hemopoietic and lymphoid tissues, other iatrogenic immunodeficiency-associated lymphoproliferative disorder (OIIA-LPD) is categorized as an immunodeficiency-related lymphoproliferative disorder. It is associated with anti-rheumatoid arthritis drugs including methotrexate (MTX), tacrolimus (TAC), and biological disease-modifying anti-rheumatic medication such as anti-TNFα drugs (1). B-cell lymphoma and Hodgkin lymphoma comprise most OIIA-LPDs, whereas T-cell lymphoma or natural killer (NK)/T-cell lymphoma comprise between 4 and 8% only (1–5). To date, only 50 patients (men, *n* = 26; women, *n* = 24) with MTX-associated T-LPDs (MTX T-LPDs) have been reported, including our patient, as detailed in Table 1 (2, 4, 6–18). Of these, 49 patients were treated for rheumatoid arthritis (RA) and one patient was treated for polymyalgia rheumatica. Data concerning the duration of MTX usage was available for 38 patients, and the median duration was 5 years (range, 0.4–24 years). Treatment for 38 patients initially involved the withdrawal of MTX only and, of these, 35 patients improved post-MTX cessation [complete response (CR), *n* = 31; partial response (PR), *n* = 4]. Chemotherapy was the initial treatment for 12 patients and response data was recorded for 10 patients [CR, *n* = 9; progressive disease (PD), *n* = 1]. Finally, data of 48 patients were available comprising 35 patients with a CR, four patients with a PR, and nine patients with PD. In total, 10 of 48 patients relapsed or progressed after initial treatment.

**Table 1:** Clinicopathological features of methotrexate-associated T-cell lymphoproliferative disorder.

**Case (References)**	**Age/Sex**	**subtype**	**Disease site**	**Biopsy site**	**MTX duration (year)**	**EBER in tumor cell**	**EBER in background**	**EBV-DNA copy (/10^**6**^ cells)**	**First line Management**	**Second line Management**	**SR**	**Response**	**Recurrence or Progression**	**Outcome (month)**
Case 1 (6)	66/F	CD30+ PC T-LPD	Skin	Skin	NA	+	NA	NA	Off MTX	–	+	CR	No	Alive
														2
Case 2 (7)	75/F	CD8+ T-LPD	Liver, spleen, LNs	LN	7	+	NA	7,400	Off MTX	Acyclovir,	-	NR → CR	No	Alive
										IVIG, steroid				7
Case 3 (8)	71/F	AITL-like	LNs	LN	20	–	+	NA	Off MTX	–	+	CR	No	NA
Case 4 (8)	68/M	AITL-like	LNs	LN	NA	–	+	NA	Off MTX	–	+	CR	No	NA
Case 5 (8)	67/M	AITL-like	LNs	LN	16	–	+	NA	Off MTX	CHOP	+	CR	Yes	NA
Case 6 (2)	60/M	AITL	LNs	LN	0.6	–	NA	NA	Off MTX	Chemotherapy	+	PR	Yes	Alive
														9
Case 7 (9)	66/F	AITL	LN	LN	0.4	–	+	290	Off MTX	Off MTX	+	CR → CR	Yes (DLBCL)	Alive
														18
Case 8 (10)	48/F	CD8+ T-LPD	Lungs, LNs, kidney, liver, spleen	LN	11.1	–	+	NA	Off MTX	–	+	PR	No	Alive
														1
Case 9 (11)	78/M	PTCL-NOS	LNs	LN	5.5	+	NA	NA	NA about MTX	–	NA	CR	No	Alive
									Chemotherapy					12
Case 10 (12)	59/F	T-LGL	BM, LN	NA	NA	–	–	NA	Off MTX	–	+	CR	No	Alive
														18
Case 11 (12)	69/F	ALK- ALCL	LN	LN	NA	–	–	NA	Chemotherapy	–	No cessation	NA	NA	Dead
														2
Case 12 (12)	61/F	T-LGL	BM, LN	NA	NA	–	–	NA	Off MTX	–	+	CR	No	Alive
														65
Case 13 (12)	70/M	AITL to T-ML, nos	LN	LN	NA	AITL-	+	NA	Off MTX	CHOP	+	CR → CR	Yes	Alive
						T-ML +								60
Case 14 (12)	31/M	SPTCL	Subcutis	Subcutis	NA	–	–	NA	Off MTX	–	+	CR	No	Alive
														24
Case 15 (4)	75/M	T-pleomorphic	NA	NA	24	NA	NA	NA	Off MTX	–	NA	NA → PD	NA	DOD
									Chemotherapy					NA
Case 16 (4)	58/M	T-LGL	NA	NA	6	NA	NA	NA	Off MTX	–	+	CR	No	Alive
														41
Case 17 (13)	77/M	PTCL-NOS	LNs	LN	22	-	NA	NA	Off MTX	CHOP	-	PD	Yes	Died for infection
														5
Case 18 (14)	60/M	SPTCL	Subcutis, abdominal cavity	Subcutis	NA	+	NA	2,000	Off MTX	–	+	CR	No	Alive
														15
Case 19 (15)	44/F	PTCL-NOS	Nasal sputum, maxillary sinus, lungs, LNs	LN	5	+	+	420	Off MTX	–	+	CR	No	Alive
														12
Case 20 (16)	66/F	CD30+ T-LPD	Lips	Lower lip	>5	+	NA	NA	Off MTX	–	+	CR	No	Alive
														12
Case 21 (17)	74/F	AITL	LNs	LN	8	-	+	NA	Off MTX	–	+	PR	No	Died for DIC
														0
Case 22 (17)	81/M	AITL	LNs	LN	NA	-	+	NA	Off MTX	–	+	CR	No	Alive
														16
Case 23 (17)	78/M	AITL	LNs	LN	5.5	-	+	NA	Off MTX	–	+	CR	No	Alive
														2
Case 24 (17)	69/M	AITL	LNs	LN	1	-	+	NA	Off MTX	–	+	PR	No	Alive with disease
														4
Case 25 (17)	75/F	AITL	LNs	LN	0.5	-	+	NA	Off MTX	NA	+	CR → PD	Yes	DOD
														49
Case 26 (17)	67/M	AITL	LNs, PB, subcutis	LN	6	-	+	NA	Off MTX	–	+	CR	No	Alive
									CHOP					31
Case 27 (17)	66/F	AITL	LNs, BM	LN	2.5	-	+	NA	Off MTX	NA	+	CR → PD	Yes (DLBCL)	DOD
														93
Case 28 (17)	72/F	AITL, EBV+ B-LPD	LNs, skin, spleen	LN, skin	NA	-	+	NA	Off MTX	–	-	CR	No	Alive
									R-CHOP					6
Case 29 (17)	57/M	AITL	LNs	LN	5	-	-	NA	Off MTX	–	+	CR	No	Alive
														45
Case 30 (17)	75/M	AITL	LNs	LN	2.4	-	+	NA	Off MTX	–	+	CR	No	Alive
									Sobuzoxane + ETO					45
Case 31 (17)	69/M	AITL with EBV+HRS	LNs	LN	21	-	+	NA	Off MTX	–	-	CR	No	Alive
									CHOP					37
Case 32 (17)	67/F	AITL	LNs, PB	LN	4.5	-	+	NA	Off MTX	–	-	CR	No	Alive
									R-CHOP					17
Case 33 (17)	79/M	AITL with EBV+HRS	LNs	LN	2.5	-	+	NA	Off MTX	NA	-	PD	-	DOD
									COP					2
Case 34 (17)	79/M	AITL	LNs	LN	15.5	-	+	NA	THP-COP	–	No cessation	CR	No	Alive
														9
Case 35 (17)	85/M	AITL with EBV+HRS	LNs	LN	0.9	-	+	NA	Off MTX	NA	-	NR → PD	-	DOD
														1
Case 36 (17)	70/M	AITL	LNs, Extranodal >1	LN	18.4	-	+	NA	Off MTX	–	+	CR	No	Alive
														68
Case 37 (17)	76/M	AITL	LNs	LN	1.8	-	+	NA	Off MTX	–	+	CR	No	Alive
														65
Case 38 (17)	67/F	AITL	LNs	LN	0.5	-	-	NA	CHOP	–	No cessation	CR	No	Alive
														14
Case 39 (17)	62/M	AITL	LNs, liver, spleen, adrenal gland	LN	3	-	+	NA	Off MTX	NA	+	CR → NA	Yes	Alive
														25
Case 40 (17)	63/F	PTCL-NOS of Tfh	LNs	LN	10	-	+	NA	Off MTX	NA	+	CR → PD	No	DOD
														37
Case 41 (17)	76/F	PTCL-NOS	LNs	LN	1.3	–	–	NA	Off MTX	–	+	CR	No	Alive
														27
Case 42 (17)	64/M	PTCL-NOS	LNs, ST	ST	7.7	–	+	NA	Off MTX	NA	+	CR → PD	Yes	DOD
														31
Case 43 (17)	80/F	PTCL-NOS	LN, oral mucosa	Oral mucosa	9.3	–	+	NA	Off MTX	-	+	CR	No	Alive
														12
Case 44 (17)	63/M	PTCL-NOS with EBV+ HRS	LNs	LN	2.3	–	+	NA	Off MTX	–	-	CR	No	Alive
									CHOP					21
Case 45 (17)	56/F	Cutaneous PTCL-NOS	Skin	Skin	4.3	–	+	NA	Off MTX	–	+	CR	No	Alive
														20
Case 46 (17)	72/F	CD8+ Cytotoxic T-LPD	LNs, pleural E, pericardial	LN	3	-	+	NA	Off MTX	–	+	CR	No	Alive
														66
Case 47 (17)	57/F	CD8+ Cytotoxic T-LPD	LNs, BM, spleen	BM	0.5	+	NA	NA	Off MTX	–	+	CR	No	Died for ASO and RB
														80
Case 48 (17)	65/F	CD8+ Cytotoxic T-LPD	LNs, liver, kidney	Liver	3	-	+	NA	Off MTX	–	+	CR	No	Alive
														13
Case 49 (17)	74/F	ATL lymphoma type	LNs	LN	5	-	NA	NA	Off MTX	No	+	CR → PD	Yes	DOD
														14
Present Case	73/M	AITL	LNs, BM	LN	17.8	-	+	1,700	Off MTX	–	+	CR	No	Alive
		EBV-positive B-cell LPD												20

*AITL, angioimmunoblastic T-cell lymphoma; ALCL, anaplastic large cell lymphoma; ASO, arteriosclerosis obliterans; ATL, adult t-cell lymphoma; BM, bone marrow; CR, complete response; DLBCL, diffuse large b-cell lymphoma; DOD, died of disease; E, effusion; EBV, Epstein-Barr virus; EBER; EBV-encoded small RNA; F, female; HRS, Hodgkin-Reed-Sternberg; LGL, large granular lymphocytic leukemia; LN, lymph node; LPD, lymphoproliferative disorder; M, male; MO, months; MTX, methotrexate; NA, not available; NOS, not otherwise specified; NR, no response; PC, primary cutaneous; PD, progressive disease; PR, partial response; PS, performance status; PTCL, peripheral T-cell lymphoma; RB, rectal bleeding; SR, spontaneous regression; ST, subcutaneous tissue; Tfh, follicular helper T cell; y, year*.

We encountered a 73-year-old male with a long medication history of MTX and TAC administration for the treatment of RA, who developed composite lymphomas consisting of angioimmunoblastic T-cell lymphoma (AITL) and Epstein-Barr virus-positive B-cell lymphoproliferative disorder (EBV-positive B-LPD). Considering the possibility of OIIA-LPD, we discontinued immunosuppressant therapy and undertook careful observation. Immunohistochemical test results indicated composite lymphomas, and both tumors were EBV-encoded RNA (EBER)-positive. In addition, his plasma level of EBV DNA copies was also high. He achieved CR only after immunosuppressant withdrawal. Therefore, composite lymphomas can be considered MTX-associated LPDs with different lineages and, here, we report one such type as a first case.

## Case Presentation

A 73-year-old man with a 20-year history of RA and a medication history of MTX (duration, 17.8 years), TAC (duration, 10.2 years), and prednisolone (PSL) was admitted to our hospital with a 4-day history of high fever and fatigue. On arrival, his vital signs were normal, except for his heart rate (113 beats/min) and body temperature (40.1°C). On physical examination, we observed right cervical lymphadenopathy. His blood test results are described in Table 2. His soluble IL-2 receptor (sIL-2R) level was markedly elevated (11,200 IU/mL; normal range, 145–519 IU/mL). A whole body computed tomography (CT) scan revealed bilateral cervical, subclavian, axilla, inguinal, mediastinal, portal, periaortic, and pelvic lymph node swelling. Given his medication history of immunosuppressant therapy, we assumed the possibility of an OIIA-LPD and consequently discontinued MTX and TAC. We continued PSL only and started intravenous antibiotics, and his elevated body temperature was soon resolved. On day 2, a bone marrow examination was conducted, and a cervical lymph node biopsy was performed on day 7.

**Table 2:** Patient laboratory data on admission.

**Complete blood count**	
White blood cells	7,300 /μL
Red blood cells	450 ×10^4^ /μL
Hemoglobin	12.3 g/dL
Hematocrit	41.2 %
Platelet	16.8 /μL
**Biochemistry**	
Total protein	6.0 g/dL
Total bilirubin	0.6 mg/dL
Albumin	2.8 g/dL
AST	36 IU/L
ALT	12 IU/L
γ-GTP	21 IU/L
LDH	365 IU/L
ALP	207 IU/L
CPK	49 IU/L
Blood urea nitrogen	27.7 mg/dL
Creatinine	1.0 mg/dL
Amylase	28 IU/L
C-reactive protein	13.0 mg/dL
sIL-2R	11,200 U/mL
Ferritin	640 ng/mL
procalcitonin	1.1 ng/mL
**Immunology**	
Anti-nuclear Ab	× <40
Rheumatoid factor	48 IU/mL
**Infection**	
T-SPOT^®^.TB Assay	Negative
EBV VCA-IgG	×160
EBV VCA-IgM	× <10
EBV EA-DR IgG	× <10
EBV EA-DR IgM	× <10
EBV EBNA	×20
CMV IgG	10.0
CMV IgM	0.6
β-D gllucan	<6 pg/mL
HBs antigen	0.00 IU/mL
HCV Ab	0.13 Log IU/mL
RPR	0.0 R.U.
TPLA	0.0 T.U.
HTLV-1	Negative

*Ab, antibody; ALP, alkaline phosphatase; ALT, alanine aminotransferase; AST, aspartate aminotransferase; CMV, cytomegalo virus; CPK, creatinine phosphokinase; EA-DR, early antigen-diffuse and restricted antibody; EBNA, Epstein-Barr virus nuclear antigen antibody; HBs, hepatitis B surface; HCV, hepatitis C virus; HTLV-1, human T-cell leukemia virus type 1; LDH, lactate dehydrogenase; RPR, rapid plasma reagin test; sIL-2R, soluble interleukin-2 receptor; TPLA, Treponema pallidum latex agglutination test; VCA, virus capsid antigen; γ-GTP, γ-glutamyl transpeptidase*.

**Figure 1:**
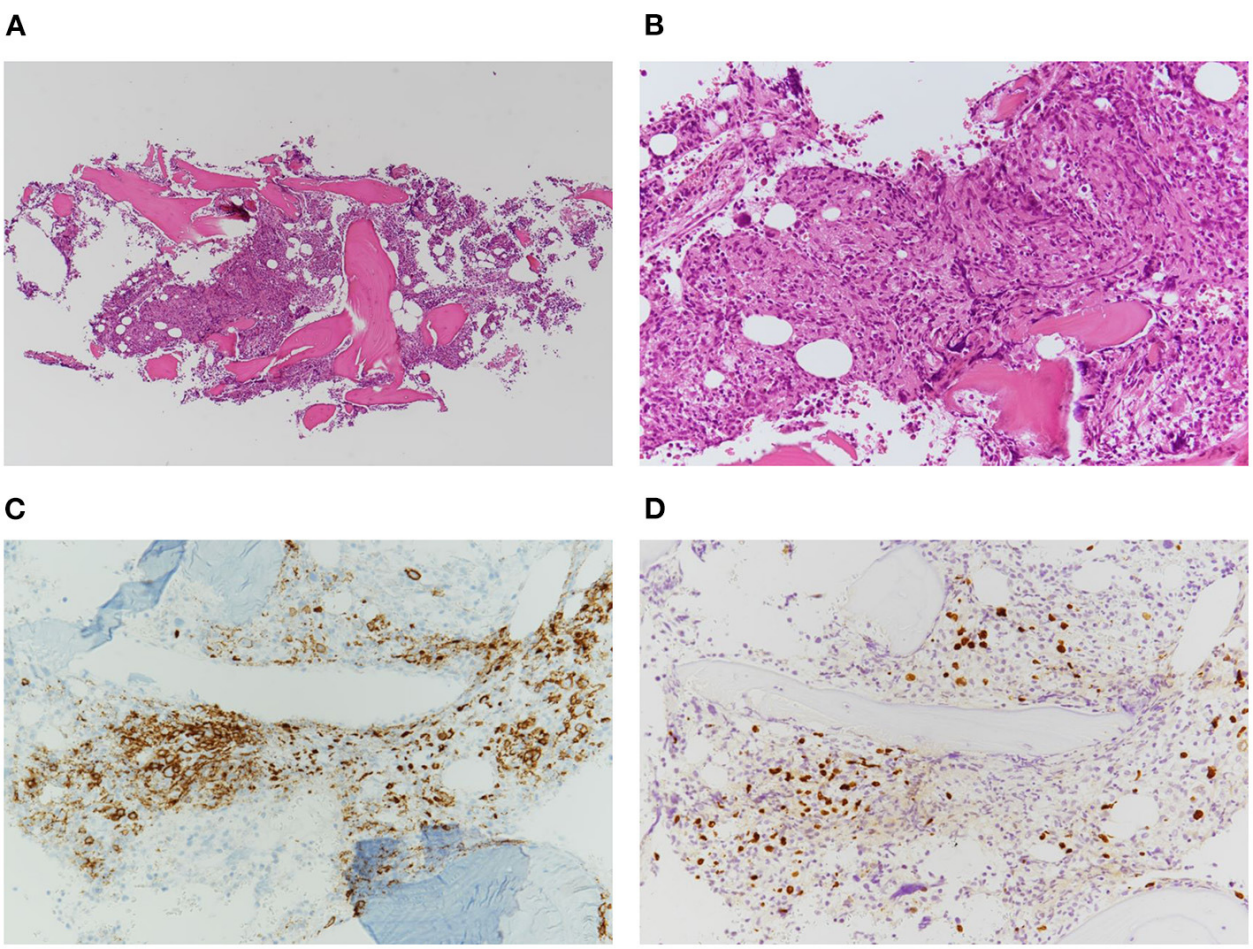
Histopathology of bone marrow biopsy showing scattered infiltration of atypical large lymphocytes. **(A)** Low-power view of the bone marrow biopsy (H&E stain, ×50). **(B)** High-power view of the atypical lymphocytes (H&E stain, ×200). **(C)** Immunohistochemical staining of CD20-positive lymphoproliferative cells (×400). **(D)** EBER *in situ* hybridization indicating positive signals in the nuclei of background cells (×200).

**Figure 2:**
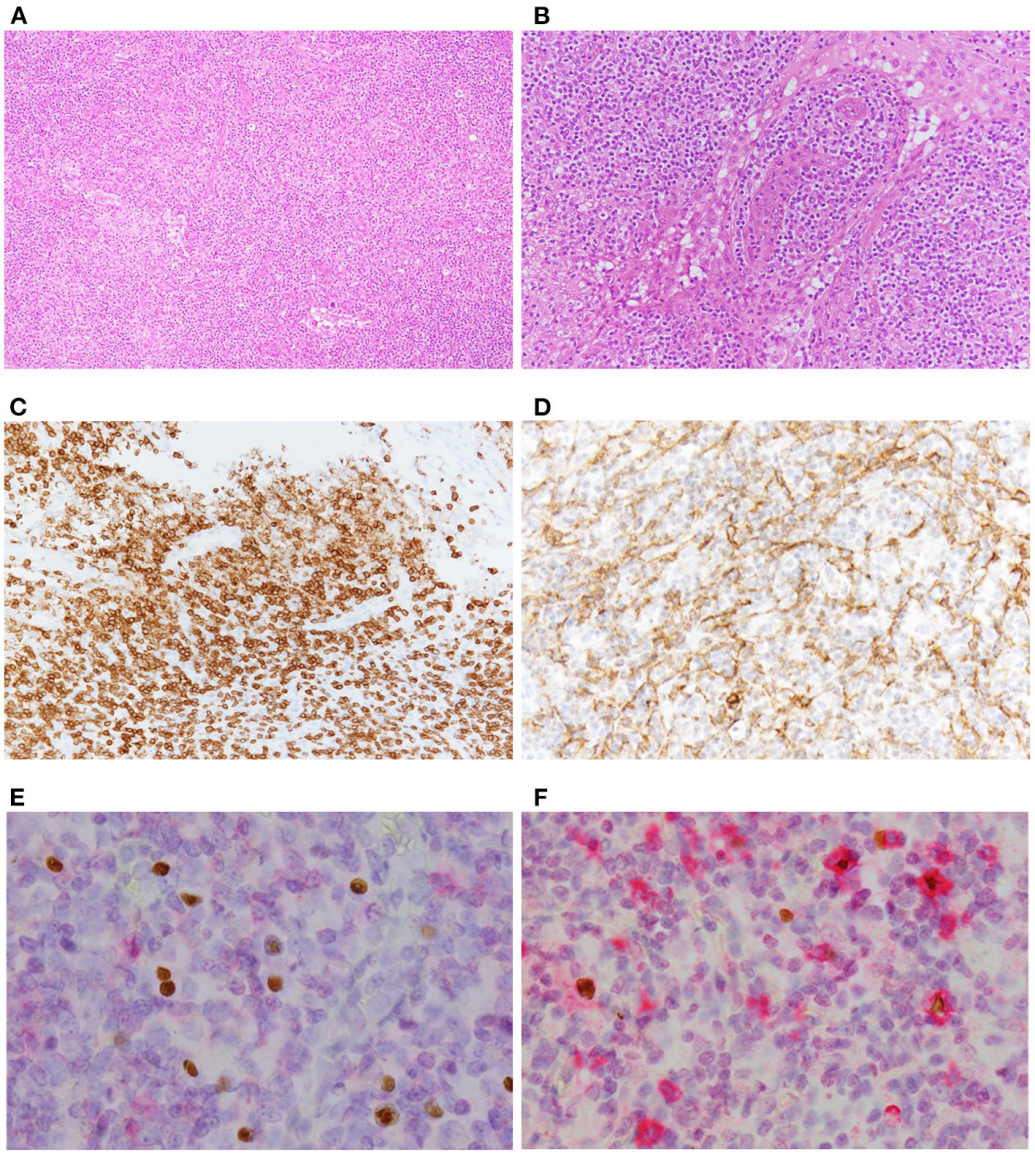
Photomicrography of the nodal biopsy. **(A)** Low-power view reveals effaced structure by marked infiltrate of small-to-medium-sized atypical lymphocytes with clear cytoplasm (H&E stain, ×100). **(B)** High-power view showing polymorphous lymphoid infiltrate with high endothelial venules (H&E stain, ×400). **(C)** Immunohistochemically, large immunoblastic lymphocytes were positive for CD20 (×400). **(D)** Podoplanin immunostain revealed expanded follicular dendritic cell meshwork (×400). **(E)** EBER *in situ* hybridization followed by PD-1 immunostaining showed that lymphoma cells were negative for EBER. **(F)** EBER *in situ* hybridization followed by the immunostaining of CD20 indicated positive-signal lymphocytes infiltrate indicating positive signals in the nuclei of background cells (×600).

Histopathological examination of the bone marrow biopsy revealed scattered infiltration of large atypical lymphocytes (Figures 1A,B). These cells were positive for CD20, CD25, and MUM1, and negative for CD3 (Figure 1C). EBER-positive lymphocytes were detected using *in situ* hybridization background staining (Figure 1D). However, histological examination of the lymph nodes showed an effaced structure with a marked increase in small-to-medium-sized atypical mononuclear cells with irregular nuclei and clear cytoplasm in a background of arborizing endothelial venules (Figures 2A,B). Immunostaining showed these atypical cells were positive for CD3 and CD4, and large immunoblastic lymphocytes scattered among the neoplastic cells were positive for CD20 (Figure 2C). No Reed-Sternberg-like cells were observed. In addition, the neoplastic cells were positive for BCL6 and CD10, suggestive of the follicular T-helper cell phenotype. Podoplanin immunostaining, a highly effective marker of follicular dendritic cells, showed an expanded follicular dendritic cell meshwork, although it was negative for CD21 (Figure 2D) (19, 20). PD-1-positive lymphocytes were EBER-negative, while CD20-positive background cells were EBER-positive (Figures 2E,F).

Diagnoses of AITL from cervical lymph nodes and of EBV-positive polymorphic B-LPD from bone marrow were confirmed. Thereafter, his lymph node swellings gradually regressed and his general condition improved. On day 22, he was discharged from hospital. Quantitative polymerase chain reaction for plasma EBV DNA on that day showed 1,700 copies/10^6^ cells (normal range, <20). On day 24, a fluorodeoxyglucose-positron emission tomography/CT scan revealed CR. His sIL-2R level dropped to 622 IU/mL on day 47, then returned to a normal level (421 IU/mL) on day 68. In addition, a bone marrow examination was conducted on day 148. Flow cytometry showed no abnormal cells, the G-band showed a normal karyotype, immunoglobulin heavy chain (IgH) rearrangement was negative, and no evidence of disease was histologically evident. Thus, he achieved CR. So far, he is still in disease-free for more than 20 months.

## Discussion

We encountered a patient with RA who had been treated with immunosuppressant therapy, who developed composite lymphomas consisting of AITL and EBV-positive B-LPD. To date, only 3 cases have been reported that have exhibited metachronous or synchronous coexistence of AITL and B-LPD as OIIA-LPDs, comprising two metachronous cases and one synchronous case (9, 17). Concerning the synchronous case, Satou et al. presented a 72-year-old woman with RA who had received MTX (17). She was diagnosed with AITL through lymph node biopsy, and was found to be EBV-positive according to cutaneous lesion biopsy results. The disease did not exhibit spontaneous regression after MTX withdrawal; therefore, she was treated with a combination of rituximab, cyclophosphamide, doxorubicin, vincristine, and prednisone and achieved CR. She remained alive 6 months after diagnosis without recurrence. Satou et al. also reported a 66-year-old woman with RA who had received MTX for 2.5 years as a metachronous case. Lymph node biopsy results were used to diagnose AITL and a CR was achieved post-MTX withdrawal. Thereafter, she relapsed and was found to have lymphadenopathy, and the lymph node biopsy results indicated diffuse large B-cell lymphoma (DLBCL). She died of the disease 93 months after initial diagnosis. Concerning a second metachronous case, Ishibuchi et al. presented a 66-year-old woman with polymyalgia rheumatica and a 4-month history of MTX therapy (9). An inguinal lymph node biopsy was performed and she was diagnosed with AITL. MTX therapy was stopped and the disease disappeared 6 months after MTX cessation. Eight months later, subcutaneous nodules appeared and a biopsy was performed, which later revealed DLBCL. With discontinuation of MTX only, the disease also regressed after 4 months. To our knowledge, no case of AITL and B-LPD simultaneously occurring and both disappearing through withdrawal of immunosuppressant therapy only has previously been reported prior to our case.

Composite lymphoma, a term introduced by Custer (21), is a rare pathological condition in which two different lymphomas co-exist simultaneously in one patient. Composite lymphoma has been reported to account for 1–4% of all lymphoma cases (22). Additionally, an analysis of 9,426 lymphoma cases in Japan revealed OIIA-LPD accounted for 147 (1.56%) cases (23). As previously stated, only 50 patients with MTX T-LPD have been reported. Composite lymphoma including T-cell lineage as an OIIA-LPD appears to be extremely rare and, as such, the clinicopathological features of MTX T-LPD remain to be elucidated. Clinicopathological feature of MTX T-LPD has yet to be elucidated because of its rarity. However, concerning our case, we consider EBV has key roles in lymphomagenesis.

In terms of EBV, Feng et al. suggested that MTX may directly reactivate latent EBV, as another cause of immunodeficiency, and lead to the development of LPDs in most MTX-associated LPDs (24). However, most proliferative T- and NK-cells are negative for EBV in MTX T-LPDs. Therefore, this suggestion does not appear readily applicable. As described in Table 1, while the tumor cells were positive for EBV in 8 (17%) of 48 patients, background cells were positive in 32 (82%) of 39 patients with available data (2, 4, 6–18). In relation to patients with AITL or those with AITL-like lymphomas, background cells were EBV-positive in 24 (96%) of 25 patients with available data. Therefore, immunodeficiency may suppress EBV-specific cytotoxic T-lymphocytes activity (25), and the reactivation of EBV suggests that the patients are immunodeficient and may suppress any immune response to prevent tumor growth. Furthermore, the relationship between EBV-positive background B-cells and AITL should be noted. AITL is a neoplasm due to clonal expansion of germinal center T-cells (26). Moreover, microarray studies have shown that tumor cells originate from follicular helper T-cells (27, 28). Of note, patients with AITL are frequently found to have EBV-positive B-cells in the microenvironment, as mentioned earlier. These B-cells accumulate somatic mutations through clonal expansion, and it has been suggested that some of these mutated cells develop B-cell lymphomas (29). Approximately 10% of patients with AITL have been found to have concurrent B-cell lymphoma at diagnosis or during the course of the disease (30, 31). In our case, B-LPD cells as well as CD20-positive cells surrounding AITL cells were EBER-positive. We speculate that MTX-associated EBV reactivation may have triggered the mutation and caused clonal expansion into the B-cells surrounding the AITL cells, leading to the development of B-LPD. Moreover, immunosuppressant can certainly accelerate lymphomageneses through inhibition of cytotoxic T-cell activity.

This case is the first to report AITL and EBV-positive B-LPD co-occurring as an OIIA-LPD that disappeared after we stopped immunosuppressant therapy only. AITL is known as a lymphoma that occasionally complicates B-LPD; thus, AITL co-locating with B-cell LPD as an OIIA-LPD might be overlooked. It is important to note that AITL can accompany B-LPD simultaneously or at a later stage, regardless of whether it is an OIIA-LPD.

## Data Availability Statement

The datasets presented in this study can be found in online repositories. The names of the repository/repositories and accession number(s) can be found in the article/supplementary material.

## Author Contributions

SK and KY wrote the manuscript, with support from all other authors. IT, JR, HA, HM, and NI treated the patient and provided the clinical history. YH and KK performed the histological examinations. All authors have critically revised and approved the final version of the manuscript.

## Conflict of Interest

The authors declare that the research was conducted in the absence of any commercial or financial relationships that could be construed as a potential conflict of interest.

## References

[1] Swerdlow SH Campo E Harris NL Jaffe ES Pileri SA Stein H WHO Classification of Tumours of Haematopoietic and Lymphoid Tissues. WHO Classification of Tumours. Revised 4th ed. Lyon: IARC Press (2017).

[2] Hoshida Y Xu JX Fujita S Nakamichi I Ikeda J Tomita Y. Lymphoproliferative disorders in rheumatoid arthritis: clinicopathological analysis of 76 cases in relation to methotrexate medication. J Rheumatol. (2007) 34:322–31. 17117491

[3] Ichikawa A Arakawa F Kiyasu J Sato K Miyoshi H Niino D. Methotrexate/iatrogenic lymphoproliferative disorders in rheumatoid arthritis: histology, Epstein-Barr virus, and clonality are important predictors of disease progression and regression. Eur J Haematol. (2013) 91:20–8. 10.1111/ejh.12116 23560463

[4] Mariette X Cazals-Hatem D Warszawki J Liote F Balandraud N Sibilia J. Lymphomas in rheumatoid arthritis patients treated with methotrexate: a 3-year prospective study in France. Blood. (2002) 99:3909–15. 10.1182/blood.V99.11.3909 12010788

[5] Yamakawa N Fujimoto M Kawabata D Terao C Nishikori M Nakashima R. A clinical, pathological, and genetic characterization of methotrexate-associated lymphoproliferative disorders. J Rheumatol. (2014) 41:293–9. 10.3899/jrheum.130270 24334644

[6] Claudino WM Gibson B Tse W Krem M Grewal J. Methotrexate-associated primary cutaneous CD30-positive cutaneous T-cell lymphoproliferative disorder: a case illustration and a brief review. Am J Blood Res. (2016) 6:1–5. 27335685 PMC4913234

[7] Hatachi S Kunitomi A Aozasa K Yagita M. CD8(+) T-cell lymphoproliferative disorder associated with Epstein-Barr virus in a patient with rheumatoid arthritis during methotrexate therapy. Mod Rheumatol. (2010) 20:500–5. 10.3109/s10165-010-0300-z 20437072

[8] Hatanaka K Nakamura N Kojima M Ando K Irie S Bunno M. Methotrexate-associated lymphoproliferative disorders mimicking angioimmunoblastic T-cell lymphoma. Pathol Res Pract. (2010) 206:9–13. 10.1016/j.prp.2009.03.005 19628340

[9] Ishibuchi H Motegi S Yamanaka M Amano H Ishikawa O. Methotrexate-associated lymphoproliferative disorder: sequential development of angioimmunoblastic T-cell lymphoma-like lymphoproliferation in the lymph nodes and diffuse large B-cell lymphoma in the skin in the same patient. Eur J Dermatol. (2015) 25:361–2. 10.1684/ejd.2015.2582 26105783

[10] Koji H Yazawa T Nakabayashi K Fujioka Y Kamma H Yamada A. CD8-positive T-cell lymphoproliferative disorder associated with Epstein-Barr virus-infected B-cells in a rheumatoid arthritis patient under methotrexate treatment. Mod Rheumatol. (2016) 26:271–5. 10.3109/14397595.2013.850613 24386983

[11] Kojima M Itoh H Hirabayashi K Igarashi S Tamaki Y Murayama K. Methtrexate-associated lymphoproliferative disorders. A clinicopathological study of 13 Japanese cases. Pathol Res Pract. (2006) 202:679–85. 10.1016/j.prp.2006.05.007 16859835

[12] Kondo S Tanimoto K Yamada K Yoshimoto G Suematsu E Fujisaki T. Mature T/NK-cell lymphoproliferative disease and Epstein-Barr virus infection are more frequent in patients with rheumatoid arthritis treated with methotrexate. Virchows Arch. (2013) 462:399–407. 10.1007/s00428-013-1389-1 23494713

[13] Miyazaki T Fujimaki K Shirasugi Y Yoshiba F Ohsaka M Miyazaki K. Remission of lymphoma after withdrawal of methotrexate in rheumatoid arthritis: relationship with type of latent Epstein-Barr virus infection. Am J Hematol. (2007) 82:1106–9. 10.1002/ajh.21003 17654684

[14] Nemoto Y Taniguchi A Kamioka M Nakaoka Y Hiroi M Yokoyama A. Epstein-Barr virus-infected subcutaneous panniculitis-like T-cell lymphoma associated with methotrexate treatment. Int J Hematol. (2010) 92:364–8. 10.1007/s12185-010-0642-5 20665252

[15] Sakaguchi R Fujikawa K Okamoto M Matsuo E Matsumoto K Uchida T. A case of rheumatoid arthritis complicated with nasal septum perforation due to methotrexate-associated lymphoproliferative disorder. Intern Med. (2019) 58:3167–71. 10.2169/internalmedicine.2995-19 31292392 PMC6875446

[16] Saleh JZ Lee LH Schieke SM Hosking PR Hwang ST. Methotrexate-induced CD30(+) T-cell lymphoproliferative disorder of the oral cavity. JAAD Case Rep. (2016) 2:354–6. 10.1016/j.jdcr.2016.02.002 27626055 PMC5011174

[17] Satou A Tabata T Miyoshi H Kohno K Suzuki Y Yamashita D. Methotrexate-associated lymphoproliferative disorders of T-cell phenotype: clinicopathological analysis of 28 cases. Mod Pathol. (2019) 32:1135–46. 10.1038/s41379-019-0264-2 30952973

[18] Takajo I Umekita K Ikei Y Oshima K Okayama A. Adult T-cell leukemia/lymphoma as a methotrexate-associated lymphoproliferative disorder in a patient with rheumatoid arthritis. Intern Med. (2018) 57:2071–5. 10.2169/internalmedicine.0308-17 29491299 PMC6096007

[19] Marsee DK Pinkus GS Hornick JL. Podoplanin (D2-40) is a highly effective marker of follicular dendritic cells. Appl Immunohistochem Mol Morphol. (2009) 17:102–7. 10.1097/PAI.0b013e318183a8e2 18838918

[20] Xie Q Chen L Fu K Harter J Young KH Sunkara J. Podoplanin (d2-40): a new immunohistochemical marker for reactive follicular dendritic cells and follicular dendritic cell sarcomas. Int J Clin Exp Pathol. (2008)1:276–84. 18784810 PMC2480560

[21] Custer R Pitfalls om the diagnosis of lymphoma and leukemia from the pathologist's point of view. In: *Second National Cancer Conference*. New York, NY (1954).

[22] Thirumala S Esposito M Fuchs A. An unusual variant of composite lymphoma: a short case report and review of the literature. Arch Pathol Lab Med. (2000) 124:1376−8. 10975943 10.5858/2000-124-1376-AUVOCL

[23] Muto R Miyoshi H Sato K Furuta T Muta H Kawamoto K. Epidemiology and secular trends of malignant lymphoma in Japan: analysis of 9426 cases according to the World Health Organization classification. Cancer Med. (2018) 7:5843–58. 10.1002/cam4.1805 30311404 PMC6247037

[24] Feng WH Cohen JI Fischer S Li L Sneller M Goldbach-Mansky R. Reactivation of latent Epstein-Barr virus by methotrexate: a potential contributor to methotrexate-associated lymphomas. J Natl Cancer Inst. (2004) 96:1691–702. 10.1093/jnci/djh313 15547182

[25] Landais E Saulquin X Houssaint E. The human T cell immune response to Epstein-Barr virus. Int J Dev Biol. (2005) 49:285–92. 10.1387/ijdb.041947el 15906243

[26] Attygalle A Al-Jehani R Diss TC Munson P Liu H Du MQ. Neoplastic T cells in angioimmunoblastic T-cell lymphoma express CD10. Blood. (2002) 99:627–33. 10.1182/blood.V99.2.627 11781247

[27] Piccaluga PP Agostinelli C Califano A Carbone A Fantoni L Ferrari S. Gene expression analysis of angioimmunoblastic lymphoma indicates derivation from T follicular helper cells and vascular endothelial growth factor deregulation. Cancer Res. (2007) 67:10703–10. 10.1158/0008-5472.CAN-07-1708 18006812

[28] de Leval L Rickman DS Thielen C Reynies A Huang YL Delsol G. The gene expression profile of nodal peripheral T-cell lymphoma demonstrates a molecular link between angioimmunoblastic T-cell lymphoma (AITL) and follicular helper T (TFH) cells. Blood. (2007) 109:4952–63. 10.1182/blood-2006-10-055145 17284527

[29] Brauninger A Spieker T Willenbrock K Gaulard P Wacker HH Rajewsky K. Survival and clonal expansion of mutating forbidden (immunoglobulin receptor-deficient) epstein-barr virus-infected b cells in angioimmunoblastic t cell lymphoma. J Exp Med. (2001) 194:927–40. 10.1084/jem.194.7.927 11581315 PMC2193480

[30] Suefuji N Niino D Arakawa F Karube K Kimura Y Kiyasu J. Clinicopathological analysis of a composite lymphoma containing both T- and B-cell lymphomas. Pathol Int. (2012) 62:690–8. 10.1111/j.1440-1827.2012.02858.x 23005596

[31] Willenbrock K Brauninger A Hansmann ML. Frequent occurrence of B-cell lymphomas in angioimmunoblastic T-cell lymphoma and proliferation of Epstein-Barr virus-infected cells in early cases. Br J Haematol. (2007) 138:733–9. 10.1111/j.1365-2141.2007.06725.x 17672882

